# On the creation of a photon by an electromagnetic wave ball

**DOI:** 10.1038/s41598-023-43757-9

**Published:** 2023-10-03

**Authors:** Gregory L. Light

**Affiliations:** Providence College, Providence, RI 02918 USA

**Keywords:** Mathematics and computing, Physics

## Abstract

Recent literature has shown, theoretically as well as experimentally, that while a beam splitter does not split a single photon, it nonetheless divides the electromagnetic wave into transmitted and reflected, with both containing energies. This implies the existence of a spacetime of pure electromagnetic waves of energies but without particles; also, it prompts the question on how much energy a photon loses after coming out of a beam splitter, which impacts on interferometry and hence quantum communication. This paper shows that, by Gauss divergence theorem, the gravitational force inside an electromagnetic wave ball results in a point energy that is three times as the wave energy; thus, a 50/50 beam splitter is to cause a photon to lose half of a quarter, or 1/8, of its initial wave energy.

## Introduction

**Figure 1:**
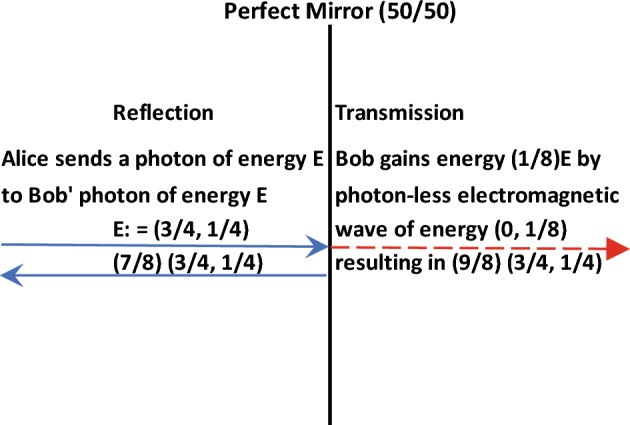
A perfect mirror to transmit a particle-less wave of energy.

The Planck formula $E = h\nu$ has rendered an identification among particle, energy, probability and wave, in short, $particle \equiv wave$ as one inseparable energy entity that is subject to Bohr’s principle of complementarity (Ref.^1^, for a summary of experimental results on the behavior of photons with respect to the quantum foundation). Through extensive literature research we have obtained, however, a limited amount of treatises that lead to a breakdown of the above identity, most notably, the construct of a beam splitter: It splits the wave but not the particle^2,3^, leaving one of the two branched-out waves devoid of the particle. The object of study of this paper is whether this particle-less wave contains energy and any associated quantification thereof. If yes^2^, then any interferometric setup for quantum communication must consider the energy losses due to the split waves, a phenomenon that research laboratories have well observed but have overall relegated to quantum entanglement^4^. Yet theoretical derivations in Ref.^5^ have shown that the Hamiltonian from the Maxwell equations has the electromagnetic field energy split $50/50$ across a perfect mirror (Ref.^6^ for an actual design) but with the photon reflected with $100\%$ probability, which apparently would incur a loss of the energy that has been transmitted. In this paper, we will present an argument to show that the energy of a photon can be decomposed into that is attributed to the point particle and that is attributed to the wave by a ratio of $3:1$. Then a ${{50} \mathord{\left/ {\vphantom {{50} {50}}} \right. \kern-0pt} {50}}$ beam splitter would cause a photon to lose energy of $\frac{1}{4} \times \frac{1}{2} = 12.5\%$, and the remainder $87.5\%$ gets redistributed by the same ratio $3:1$ into $87.5\% \times \frac{3}{4} + 87.5\% \times \frac{1}{4}$ (particle and wave, respectively). That is, a ${{50} \mathord{\left/ {\vphantom {{50} {50}}} \right. \kern-0pt} {50}}$ perfect mirror splits $\frac{1}{2}$ of the wave energy $\frac{E}{4}$ from the incident photon $\left( {{\text{particle of }}\frac{3E}{4},\;{\text{wave of }}\frac{E}{4}} \right)$ into a reflected photon of a reduced energy $\frac{7E}{8}$, i.e., $\left( {{\text{particle of }}\frac{7}{8} \cdot \frac{3E}{4},\;{\text{wave of }}\frac{7}{8} \cdot \frac{E}{4}} \right)$, and a photon-less transmitted electromagnetic wave, $\left( {0,\;{\text{wave of }}\frac{E}{8}} \right)$ (see Fig. 1), which if absorbed by other surrounding particles, we identify as a virtual photon, and otherwise, dark energy, to observe conservation of energy. The reason for the reflected photon to maintain the same energy ratio $3:1$ over particle and wave as before the incidence is that a ball of wave energy, by Gauss divergence theorem, produces a particle of threefold energy, which is our main result to be shown below. Since the photon-less wave nevertheless contains energy, it effects quantum counterfactual communication^7,8^.

Our derivation below will basically show that a spinning “electromagnetic wave ball” (to be defined in the ensuing analysis) makes a vortex with the center as a sink, giving out a point energy, wherein the dynamics is governed by the negative potential energy from the gravitational radial attraction plus the positive kinetic energy for the spinning motion equal to zero, and wherein Gauss divergence theorem takes the form of the integral of the divergence of the gravitational force inside the ball equal to a point measure—the angular momentum—of the ball center that serves as the boundary between the wave spacetime of the electromagnetic wave ball and the particle spacetime for the created photon (cf. Ref.^9^ for quantum vacuum fluctuations in front of a perfect mirror). We note that the divergence theorem has in its assumptions a compact domain, so that not all electromagnetic waves carry photons—exactly the point that we are making. The rest of this paper is organized as follows: In the main analysis below, we will first set up a spacetime frame for an isolated electromagnetic wave ball, next calculate the divergence of the gravitational force within the ball, and then conclude that the wave energy inside the ball produces a particle of three times its energy. Finally, we present a summary citing the only existing experimental confirmation of our result to date, sketching a new approach to quantum counterfactual communication, and presenting a potential interest of study.

## Analysis

By Maxwell Equations, an electromagnet wave traveling in the z-direction with an angular frequency $\omega$ in a reference frame can be represented as follows,

1 $$\begin{aligned} & {\mathbf{E}} + {\mathbf{B}} + {\mathbf{S}} \in {\mathbb{R}}_{{\left( {x,y,z} \right)}}^{3} \\ & : = \left( {\left\| {\mathbf{E}} \right\|_{\max } \cos \left( {kz - \omega t} \right),\frac{1}{c}\left\| {\mathbf{E}} \right\|_{\max } \cos \left( {kz - \omega t} \right),c\varepsilon_{o} \left\| {\mathbf{E}} \right\|_{\max }^{2} \cos \left( {kz - \omega t} \right)} \right), \\ \end{aligned}$$

where ${\mathbf{E}} \equiv$ the electric field, ${\mathbf{B}} \equiv$ the magnetic field, ${\mathbf{S}} \equiv$ the Poynting vector, and $\varepsilon_{o} \equiv {\text{permittivity constant}}$. Associated with this “electromagnetic disturbance is a mass;” by its internal gravity a “spherical geon” can result^10^, which we will call “an electromagnetic wave ball.”

Let a laboratory reference frame

2 $$\Phi : = \left\{ \begin{aligned} & \left( {t,x,y,z} \right) \in {\mathbb{R}}^{1 + 3} \left| {\left\| {\left( {\Delta t,\Delta x,\Delta y,\Delta z} \right)} \right\|_{Minkowski}^{2} } \right. \\ & : = c^{2} \Delta t^{2} - \Delta x^{2} - \Delta y^{2} - \Delta z^{2} \equiv c^{2} \Delta t_{0}^{2} \\ \end{aligned} \right\}$$

be given, in which there is an isolated electromagnetic wave ball $B$ of radius $R$ containing energy $E_{B} = M_{B} c^{2}$. Then the “proper time” $\Delta t_{0}$ as defined in Eq. (2) applies to the center $O_{B}$ of $B$ that in itself is set at a state of rest; call this frame $\Phi_{0}$, wherein $t_{0} = 1\;$ is defined to be the duration for $B$ to spin counterclockwise around the $z - axis$ for exactly one cycle, and $it_{0} : = {{t_{0} } \mathord{\left/ {\vphantom {{t_{0} } {2\pi }}} \right. \kern-0pt} {2\pi }}$ is defined to be the duration for $B$ to spin for exactly $1$ radian, with the fine distinction that $it_{0} \in S^{1}$ is in a (circular) quotient space and $t_{0} \in {\mathbb{R}}$ is in a (linear) Cartesian dimension. For either expression, the measure is relativistically invariant since the Planck constant $h$ is universal and so must $E = h \cdot 1 \equiv h \cdot \left( {{1 \mathord{\left/ {\vphantom {1 {t_{0} }}} \right. \kern-0pt} {t_{0} }}} \right)$ be (albeit the time *second* in any reference frame to observe $k\;cycles$, or, frequency, is not). By the definition of $it_{0} : = {{t_{0} } \mathord{\left/ {\vphantom {{t_{0} } {2\pi }}} \right. \kern-0pt} {2\pi }}$, one has

3 $${\text{spatial distance }} \equiv {\text{ time duration,}}$$

so that relative to $O_{B}$, any point $p \in B - \left\{ {O_{B} } \right\}$ has its motion of unit speed, thereof a constant velocity gravitating radially toward $O_{B}$ or a circular motion of

4 $$re^{{it_{0} }} \equiv 1 \cdot e^{{it_{0} }} ,{\text{ implying that, in particular, }}\left| {\frac{{d^{2} }}{{d\left( {it_{0} } \right)^{2} }}e^{{it_{0} }} } \right| = \left| { - e^{{it_{0} }} } \right| = 1 \, .$$

In passing, since the motion $c\left( {t_{0} } \right)$ has a unit speed, one has:

5 $$\left( {kinetic} \right)\;energy\;E_{B} = h\nu = h = mass\;M_{B} \cdot c^{2} = M_{B} = p_{B} c = momentum\;p_{B} .$$

To prepare for our main result below, we must first establish the claim that $E_{B}$ is uniformly continuously distributed over $B$. Here, by gravitational isotropy the energy contained in any sphere $S_{r}^{2} \subset B,\;r \in \left[ {0,R} \right]$, is uniformly distributed over $4\pi r^{2}$. It remains to show that if $p_{1} \in S_{{r_{1} }}^{2}$ and $p_{2} \in S_{{r_{2} }}^{2}$ with $r_{1} < r_{2}$, then $p_{1}$ and $p_{2}$ nevertheless have the same energy. Here:

Let $p_{\alpha } = \left( {0,0,0,z_{\alpha } } \right) \in B$ in $\Phi_{0}$. By Maxwell equations, $p_{\alpha }$ has energy $\varepsilon_{o} \left\| {\mathbf{E}} \right\|_{\max }^{2} \cdot \left| {\cos \left( {kz_{\alpha } - \omega t_{o} } \right)} \right|$ over $t_{o} \in \left[ {0,1} \right]$, so that at $t_{o} = 1$, $p_{\alpha }$ accumulates an energy of

6 $$\begin{aligned} & \varepsilon_{o} \left\| {\mathbf{E}} \right\|_{\max }^{2} \int_{0}^{1} {\left| {\cos \left( {kz_{\alpha } - \omega t_{o} } \right)} \right|} \;dt_{o} = \varepsilon_{o} \left\| {\mathbf{E}} \right\|_{\max }^{2} \int_{0}^{2\pi } {\left| {\cos \theta } \right|} \;d\theta \\ & = 4\varepsilon_{o} \left\| {\mathbf{E}} \right\|_{\max }^{2} \int_{0}^{{\frac{\pi }{2}}} {\cos \theta } \;d\theta = 4\varepsilon_{o} \left\| {\mathbf{E}} \right\|_{\max }^{2} . \\ \end{aligned}$$

Since $z_{\alpha } \in \left[ { - R,R} \right]$ is arbitrary, $p_{1}$ and $p_{2}$ have the same energy. Therefore, every $p \in B$ has the same energy $\rho_{p} \equiv {{E_{B} } \mathord{\left/ {\vphantom {{E_{B} } {\left( {\frac{{4\pi R^{3} }}{3}} \right)}}} \right. \kern-0pt} {\left( {\frac{{4\pi R^{3} }}{3}} \right)}}$.

**Figure 2:**
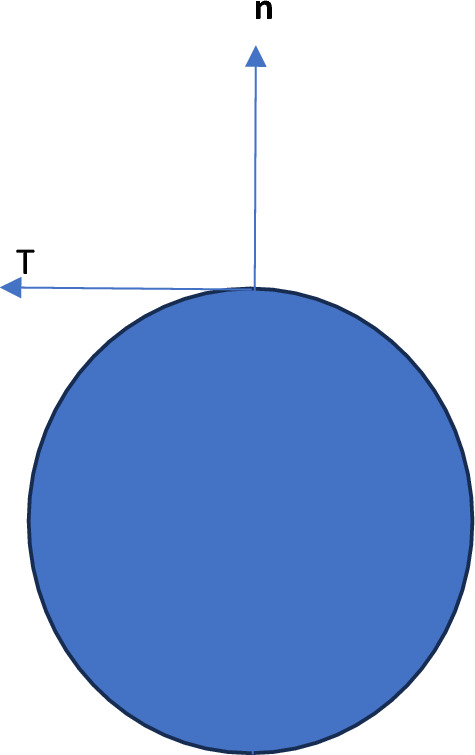
Any energy point in ball B to follow a geodesic motion, to stay on the same plane spanned by n and T.

To prepare for our main result below, we must also establish one more claim, that for any $p \in B - \left\{ {O_{B} } \right\}$ the motion of $p$ is $re^{{\left( { - 1 + i \cdot 2\pi } \right)t_{0} }}$ (cf. Ref.^11^ for quantum gravity in blackholes with logarithmic transformation); i.e., as $p$ spins by one cycle, $p$ moves radially toward $O_{B}$ by $r \cdot \left( {1 - \frac{1}{e}} \right)$. Here, the energy of $p$ is established by the spin of $B$ exactly by one cycle. For the same duration of time, $p$ is also the potential energy released in its motion from $r$ to $\frac{r}{e}$, as shown below. Since the spacetime of $B$ of energy $h \cdot 1 \approx 0$ is effectively flat, we apply Newton’s law of gravitation,

7 $$- \int_{{{r \mathord{\left/ {\vphantom {r e}} \right. \kern-0pt} e}}}^{r} {\frac{d}{{ds_{0} }}\left( {\frac{{GM_{B} \rho_{p} }}{{s_{0} }}} \right)} \;ds_{0} = \int_{{{r \mathord{\left/ {\vphantom {r e}} \right. \kern-0pt} e}}}^{r} {\frac{{GM_{B} \rho_{p} }}{{s_{0}^{2} }}} \;ds_{0} = \int_{{{r \mathord{\left/ {\vphantom {r e}} \right. \kern-0pt} e}}}^{r} {a\rho_{p} } \;ds_{0} ,$$

where

8 $$a = - 1{ ,}$$

 with the distance measured by $$r = \left\| {\overset{\lower0.5em\hbox{$\smash{\scriptscriptstyle\rightharpoonup}$}}{{O_{B} p_{\alpha } }} } \right\|$$ as from Eq. (3) (For a formal treatment, see Appendix, where the detailed motion of $$p$$, $$re^{{\left( { - 1 + i \cdot 2\pi } \right)t_{o} }}$$, is ascertained by the condition of it being a geodesic; i.e., $$p$$ must stay on the plane as spanned by its radius $$\overset {\rightarrow}r$$ and its tangent as shown in Fig. 2).

Thus, the motion of $p$ is: $re^{{\left( { - 1 + i \cdot 2\pi } \right)t_{o} }}$(cf. Ref.^12^ for exponential mapping in relation to geodesics), which can be summarized by the following pair of differential equations:

9 $$\frac{{d\ln \left( {r\left( {t_{o} } \right)} \right)}}{{dt_{o} }} = - 1\quad {\mathrm{and}}\quad \frac{{d\left( {\theta \left( {t_{o} } \right)} \right)}}{{dt_{o} }} = 2\pi i.$$

We now consider a decomposition of $M_{B} : = M_{w} + M_{p} {\text{, with }}M_{w} = \omega M_{B} ,\;\omega \in \left( {0,1} \right]$, allowing for $M_{B} = M_{w} \left( { \Leftrightarrow M_{p} = 0} \right)$, and compute the divergence of the gravitational force inside $B$ *at the completion of one cycle*. Here, since $M_{B}$ is determined by the radius $R$ (as from $h\nu = \frac{hc}{\lambda } = \frac{hc}{{2R}}$), it is the gravitational motion of the mass $\left\{ {p\left| {p \in S_{R}^{2} } \right.} \right\}$ on the ball boundary that calculates the gravitational force in $B$; with Eq. (7) being frame invariant, we thus have:

10 $$\begin{aligned} & \iiint_{B} {div\left( {\frac{{\omega M_{B} }}{{{{4\pi R^{3} } \mathord{\left/ {\vphantom {{4\pi R^{3} } 3}} \right. \kern-0pt} 3}}}} \right)}\left( {\frac{{GM_{B} }}{{r^{2} }}} \right) \cdot \frac{{\mathbf{r}}}{{\left\| {\mathbf{r}} \right\|}}dxdydz \\ & = \left( {\frac{{M_{B} }}{{{{4\pi R^{3} } \mathord{\left/ {\vphantom {{4\pi R^{3} } 3}} \right. \kern-0pt} 3}}}} \right)R^{3} \int_{{{1 \mathord{\left/ {\vphantom {1 e}} \right. \kern-0pt} e}}}^{1} {\left( {\iint_{{S_{r}^{2} }} {\frac{d}{dr}\left( {\frac{{G\omega M_{B} }}{{r^{2} }}} \right)d\sigma }} \right)} dr \\ & = \left( {\frac{{M_{B} }}{{{{4\pi R^{3} } \mathord{\left/ {\vphantom {{4\pi R^{3} } 3}} \right. \kern-0pt} 3}}}} \right)R^{3} \int_{{{1 \mathord{\left/ {\vphantom {1 e}} \right. \kern-0pt} e}}}^{1} { - \frac{{2G\omega M_{B} }}{{r^{3} }}} \cdot 4\pi r^{2} dr \\ & = - \left( {\frac{{M_{B} }}{{{{4\pi R^{3} } \mathord{\left/ {\vphantom {{4\pi R^{3} } 3}} \right. \kern-0pt} 3}}}} \right)R^{3} \cdot 8\pi G\omega M_{B} \int_{{{1 \mathord{\left/ {\vphantom {1 e}} \right. \kern-0pt} e}}}^{1} \frac{1}{r} \;dr \\ & = - \frac{3}{4}M_{B} \cdot 8G\omega M_{B} = - \frac{3}{4}M_{B} \cdot c \cdot \frac{{8G\omega M_{B} }}{{c^{2} }},\quad \left( {c = 1} \right), \\ & = - \frac{3}{4}M_{B} \cdot c \cdot R_{Schwarzschild} \Leftrightarrow \omega = \frac{1}{4}, \\ \end{aligned}$$

where the negative sign is due to the direction of the integration from $S_{{{R \mathord{\left/ {\vphantom {R e}} \right. \kern-0pt} e}}}^{2} {\text{ to }}S_{R}^{2}$. For later references, denote by ${\mathbf{H}}$

11 $$\begin{aligned} & - \iiint_{B} {div\left( {\frac{{\omega M_{B} }}{{{{4\pi R^{3} } \mathord{\left/ {\vphantom {{4\pi R^{3} } 3}} \right. \kern-0pt} 3}}}} \right)}\left( {\frac{{GM_{B} }}{{r^{2} }}} \right) \cdot \frac{{\mathbf{r}}}{{\left\| {\mathbf{r}} \right\|}}dxdydz \\ & = \frac{3}{4}M_{B} \cdot c \cdot R_{Schwarzschild} \equiv {\mathbf{H}}. \\ \end{aligned}$$

In the above, $\frac{{2GM_{B} }}{{c^{2} }} = R_{Schwarzschild}$ is because the interior of $B$ containing $M_{w}$ has periodic or imaginary spacetimes (cf. Ref.^13^); i.e., by Einstein field equations, for all $r \in \left( {0,R_{Schwarzschild} } \right)$ one has $g_{11} = 1 - \frac{{2GM_{B} }}{{rc^{2} }} = \left( {\frac{{\Delta t_{o} }}{\Delta t}} \right)^{2} < 0$ relative to $\, \Delta t$ in $\Phi$. As such,

12 $$M_{w} = \frac{1}{4}M_{B} \;{\mathrm{and}}\;M_{p} = \frac{3}{4}M_{B} .$$

In summary, the solution to Eq. (10)—*energy added into *$B$* due to the gravitational field produced by *$M_{w}$ equals *the angular momentum of* $M_{p}$,

13 $$\begin{aligned} & {\mathbf{\rm H}} \equiv - \iiint_{B} {\rho \cdot div\left( {\frac{{G \cdot \left( {M_{w} + M_{p} } \right)}}{{r^{2} }}} \right) \cdot \frac{{\mathbf{r}}}{{\left\| {\mathbf{r}} \right\|}}d{\mathbf{x}}} \\ & = M_{p} \cdot c \cdot R_{Schwarzschild} = {\text{the angular momentum of }}M_{p} , \\ \end{aligned}$$

is obtained for $M_{p} = \frac{3}{4}M_{B}$, where the effect of $M_{p}$ in the integrand is to have $M_{p}$ traveling at the expectation speed of $c$ in the $z - direction$ of angular momentum ${\mathbf{H}}$, i.e., a photon with its associated electromagnetic wave mass $M_{w}$ in $B$ spiraling toward the sink $O_{B}$ cycle after cycle (cf. Ref.^14^ for Poynting vectors taking on complex values).

We note that the periodicity of the cosine function in Eq. (1) implies that as $\theta = - \frac{\pi }{2}$ turns to $0$ and continues to $\frac{\pi }{2}$, each point $p_{\alpha } \in B$ has its energy $\rho_{p} \left( {t_{0} } \right) = \varepsilon_{o} \left\| {\mathbf{E}} \right\|_{\max }^{2} \cdot \left| {\cos \left( {kz_{\alpha } - \omega t_{0} \equiv \theta } \right)} \right|$ rising from $0$ to $\varepsilon_{o} \left\| {\mathbf{E}} \right\|_{\max }^{2}$ and then falling to $0$, for *one energy cycle*, but this leaves the energy from the remaining $\left[ {\frac{\pi }{2} \to \pi \to \frac{ - \pi }{2}} \right]$ unaccounted for. As such, in measuring the angular momentum of $B$, which entails $2\pi$ radians, one must include this “residual” energy $\Delta E = h\nu = h$; i.e., $\left[ {0,\lambda } \right]$ contains $2B^{\prime}s$. Thus,

$$\begin{aligned} & {\mathbf{\rm H}} \equiv M_{p} \cdot c \cdot R_{Schwarzschild} = \frac{3}{4}M_{B} \cdot 2GM_{B} \quad \cdots \left( * \right) \\ & = \frac{3}{4}M_{B} \cdot 2G \cdot \left( {\frac{h\nu + \Delta E}{{c \cdot 2R_{Schwarzschild} \nu }}} \right) \\ \end{aligned}$$

$$\left( {{\text{where }}M_{B} = \frac{{E_{B} }}{{c^{2} }} = \frac{2h\nu }{{c \cdot \lambda \nu }} = \frac{2h\nu }{{c \cdot 2R_{Schwarzschild} \nu }}} \right)$$

$$= \frac{3}{4}M_{B} \cdot 2G\left( {\frac{h}{c} \cdot \frac{{c^{2} }}{{2GM_{B} }}} \right) = \frac{3}{4}M_{B} \cdot 2G\left( {\frac{hc}{{2GM_{B} }}} \right)\quad \cdots \left( {**} \right)$$

$$\left( {{\text{where }}\frac{hc}{{2GM_{B} }} = M_{B} ,{\text{ by comparing }}\left( {*} \right){\text{ with }}\left( {**} \right),{\text{ i}}{\mathrm{.e}}{., }M_{B}^{2} { = }\frac{hc}{{2G}}} \right){,}$$

$$\begin{gathered} = \frac{3}{4} \cdot hc = \frac{3}{4}h = M_{p} = \frac{3}{4}M_{B} ; \hfill \\ {\mathrm{i}}{\mathrm{.e}}{.,}\,M_{B} = p_{B} = h,{\text{ as is known}}{.} \hfill \\ \end{gathered}$$

## Summary

An experiment in ^15^ (Appendix D, ibid.) had a ${{54} \mathord{\left/ {\vphantom {{54} {46}}} \right. \kern-0pt} {46}}$ beam splitter result in the measured energy of the output photon $88\%$ as before, in close agreement with our derived value, $\frac{3}{4}E + \frac{1}{4} \cdot \frac{54}{{100}}E = 0.885E$. Theoretically, our derived result can also present an explanation of the well-known electromagnetic mass of an electron of ratio $4:3$ between that is moving and that is stationary; that is, whilst the electron moves along with its wave, it is, otherwise, a point charge subject to the Coulomb law. We have also provided a network structure for the distribution of energies in an interferometric setup, wherein the loss of energy has been drawing attention from researchers^16^. As an example, consider a photon of energy $E$ incident upon two ${{50} \mathord{\left/ {\vphantom {{50} {50}}} \right. \kern-0pt} {50}}$ beam splitters in succession. Then in terms of proportions of $E$, we have (cf. Fig. 1):

$1 \equiv \left( {\frac{3}{4},\frac{1}{4}} \right)_{p,w} = \left( {\frac{3}{4} \times \frac{{\mathbf{7}}}{{\mathbf{8}}},\frac{1}{4} \times \frac{{\mathbf{7}}}{{\mathbf{8}}}} \right)_{p,w} + \left( {0,\frac{1}{8}} \right)_{w}$ after the first split;

now upon the second split:

1. The first term $\left( {\frac{3}{4} \times \frac{{\mathbf{7}}}{{\mathbf{8}}},\frac{1}{4} \times \frac{{\mathbf{7}}}{{\mathbf{8}}}} \right)_{p,w}$ splits into—
  $$\left( {\frac{3}{4} \times \left( {\frac{7}{8} \times \frac{{\mathbf{7}}}{{\mathbf{8}}}} \right),\frac{1}{4} \times \left( {\frac{7}{8} \times \frac{{\mathbf{7}}}{{\mathbf{8}}}} \right)} \right)_{p,w} + \left( {0,\frac{1}{8} \times \frac{{\mathbf{7}}}{{\mathbf{8}}}} \right)_{w} ;$$
  i.e., each split causes the photon to lose $\frac{1}{8}$ of its energy, with the retained energy decreasing in a geometric sequence, $\frac{7}{8},\;\left( \frac{7}{8} \right)^{2} , \cdots$
2. The second term $\left( {0,\frac{1}{8}} \right)_{w}$ splits into— $\left( {0,\frac{1}{16}} \right)_{w} + \left( {0,\frac{1}{16}} \right)_{w} ,$ i.e., two waves.

That is, the energy distribution of the incident photon after two ${{50} \mathord{\left/ {\vphantom {{50} {50}}} \right. \kern-0pt} {50}}$ beam splitters is:$1 \equiv \left( {\frac{3}{4},\frac{1}{4}} \right)_{p,w} = \left( {\frac{3}{4} \times \frac{49}{{64}},\frac{1}{4} \times \frac{49}{{64}}} \right)_{p,w} + \left( {0,\frac{7}{64}} \right)_{w} + \left( {0,\frac{4}{64}} \right)_{w} + \left( {0,\frac{4}{64}} \right)_{w} .$

Another implication of our result here is on quantum counterfactual communication, where information transmission is free from particles, notably, the Elitzer-Vaidman bomb test. Thus, if a particle-less wave still contains energy, then Alice can use a perfect mirror to transmit a wave energy to Bob and execute the communication (see Figs. 1, 2). One possible research interest out of the present work might be a re-examination of the relationships among the fundamental physical constants since our analysis here involves the permittivity constant.

## Supplementary Information

Supplementary Information.

## Data Availability

The datasets used and/or analysed during the current study available from the corresponding author on reasonable request.
