# Correction: On the creation of a photon by an electromagnetic wave ball

**DOI:** 10.1038/s41598-026-58317-0

**Published:** 2026-06-17

**Authors:** Gregory L. Light

**Affiliations:** Providence College, Providence, RI 02918 USA

Correction to: *Scientific Reports* 10.1038/s41598-023-43757-9, published online 03 October 2023

This Article contains errors. In Equations 1 and 6 the exponent squares are missing. Furthermore, Equation (6) is missing the multiplier of π, and contains the erroneous multiplier of ‘4’. The correct equations are stated below.

Equation 1:

$$\begin{array}{cc}& \mathbf{E}+\mathbf{B}+\mathbf{S}\in {\mathbb{R}}_{\left(x,y,z\right)}^{3}\\ & :=\left({\Vert \mathbf{E}\Vert }_{\mathrm{m}\mathrm{a}\mathrm{x}}\mathrm{cos}\left(kz-\omega t\right),\frac{1}{c}{\Vert \mathbf{E}\Vert }_{\mathrm{m}\mathrm{a}\mathrm{x}}\mathrm{cos}\left(kz-\omega t\right),c{\varepsilon}_{o}{\Vert \mathbf{E}\Vert }_{\mathrm{m}\mathrm{a}\mathrm{x}}^{2}\mathrm{cos}\left(kz-\omega t\right)\right),\end{array}$$

should read:

$$\begin{array}{cc}& \mathbf{E}+\mathbf{B}+\mathbf{S}\in {\mathbb{R}}_{\left(x,y,z\right)}^{3}\\ & :=\left({\Vert \mathbf{E}\Vert }_{\mathrm{m}\mathrm{a}\mathrm{x}}\mathrm{cos}\left(kz-\omega t\right),\frac{1}{c}{\Vert \mathbf{E}\Vert }_{\mathrm{m}\mathrm{a}\mathrm{x}}\mathrm{cos}\left(kz-\omega t\right),c{\varepsilon}_{o}{\Vert \mathbf{E}\Vert }_{\mathrm{m}\mathrm{a}\mathrm{x}}^{2}{\mathrm{cos}}^{2}\left(kz-\omega t\right)\right),\end{array}$$

Equation 6:

$$\begin{array}{cc}& {\varepsilon}_{o}{\Vert \mathbf{E}\Vert }_{\mathrm{m}\mathrm{a}\mathrm{x}}^{2}{\int}_{0}^{1}\left|\mathrm{cos}\left(k{z}_{\alpha }-\omega {t}_{o}\right)\right|d{t}_{o}={\varepsilon}_{o}{\Vert \mathbf{E}\Vert }_{\mathrm{m}\mathrm{a}\mathrm{x}}^{2}{\int}_{0}^{2\pi }\left|\mathrm{cos}\theta \right|d\theta \\ & =4{\varepsilon}_{o}{\Vert \mathbf{E}\Vert }_{\mathrm{m}\mathrm{a}\mathrm{x}}^{2}{\int}_{0}^{\frac{\pi }{2}}\mathrm{cos}\theta d\theta =4{\varepsilon}_{o}{\Vert \mathbf{E}\Vert }_{\mathrm{m}\mathrm{a}\mathrm{x}}^{2}.\end{array}$$

should read:

$$\begin{array}{cc}& {\varepsilon}_{o}{\Vert \mathbf{E}\Vert }_{max}^{2}{\int}_{0}^{1}{\left|\mathrm{cos}\left(k{z}_{\alpha }-\omega {t}_{o}\right)\right|}^{2}d{t}_{o}={\varepsilon}_{o}{\Vert \mathbf{E}\Vert }_{max}^{2}{\int}_{0}^{2\pi }{\left|\mathrm{cos}\theta \right|}^{2}d\theta \\ & =4{\varepsilon}_{o}{\Vert \mathbf{E}\Vert }_{max}^{2}{\int}_{0}^{\frac{\pi }{2}}{\mathrm{cos}}^{2}\theta d\theta =\pi {\varepsilon}_{o}{\Vert \mathbf{E}\Vert }_{max}^{2}.\end{array}$$

