# Supplementary material for: On the creation of a photon by an electromagnetic wave ball
Source: Sci Rep. 2023 Oct 3;13:16634. doi: 10.1038/s41598-023-43757-9 (PMC10547803; doi:10.1038/s41598-023-43757-9)
Supplement: Supplementary file 1 — Supplementary Information. [file 41598_2023_43757_MOESM1_ESM.docx]

**Appendix**

(Notations in the text continue below.) Let $p_{\alpha}\in B-\left\{O_{B}\right\}$; consider the motion of the unit-speed curve $c\left(t_{0}\right)$ of $p_{\alpha}$ as from equation (3), , $c\left(0\right)\equiv p_{\alpha}$. Define (see Figure 2)

, the velocity vector of $c\left(t_{0}\right)$,

$\mathbf{n}:=\overrightarrow{O_{B}p_{\alpha}}/\left\|\overrightarrow{O_{B}p_{\alpha}}\right\|$, the unit normal vector of $c\left(t_{0}\right)$, and

$S:=\mathbf{n}\times T$, the “intrinsic normal” of $c\left(t_{0}\right)$, which would be the direction for $c\left(t_{0}\right)$ to deviate from being a geodesic. I.e., $c\left(t_{0}\right)$, being a geodesic, stays within the plane spanned by $\mathbf{n} and T.$

The general condition of a geodesic $c\left(t\right)$ in an n-manifold $M^{n}$ [12] is

(14)

where and

$\Gamma_{ij}^{k}\equiv$ Christoffel symbol of the second kind the coefficient of the basis vector $\frac{\partial}{\partial x^{k}}$ in the expansion of the covariant derivative $\nabla_{\frac{\partial}{\partial x^{i}}}\frac{\partial}{\partial x_{j}}\equiv\sum_{k=1}^{n} \Gamma_{ij}^{k}\frac{\partial}{\partial x^{k}}\in T_{p}M^{n}$ the tangent space at point $p\in M^{n}$. As an illustration - -

In the case of a surface in which has been parametrized by , one seeks to solve for a unit-speed curve , in that corresponds to the curve on the surface moving always within the plane spanned by its $\mathbf{n} and T$. $\Gamma_{11}^{1 or 2}$, for example, has the physical meaning of a East-bound acceleration in the map to cause what corresponding acceleration on the surface as manifested on the map along the East or the North direction. $\Gamma_{12}^{1 or 2}$ has the physical meaning of the acceleration of a North-bound velocity due to a East-bound variation in the map to cause what corresponding acceleration on the surface as manifested on the map along the East or the North direction.

The right-hand 0’s in equation (14) are the conditions for $c\left(t\right)$ to be a geodesic.

In our present case, the “surface” is a curve $c\left(t\right)$ in and . Then , measuring the acceleration of $p_{\alpha}\left(t\right)$ along a directed line, which must be $\mathbf{n}:=\overrightarrow{O_{B}p_{\alpha}}/\left\|\overrightarrow{O_{B}p_{\alpha}}\right\|$ owing to the definition of $B$ being a geon. Then equation (14) reduces to

(15)

since $\left|\frac{d^{2}c_{T}\left(t_{0}\right)}{dt_{0}^{2}}\right|$==1 by equation (4), one has for equation (8).

The first term in equation (15) corresponds to the rotation $e^{it_{0}}$ of $c\left(t_{0}\right)$ in requiring kinetic energy and the second term corresponds to the radial motion of $c\left(t_{0}\right)$ toward $O_{B}$ releasing potential energy. For a geometric description of the flows in $B$, we partition $B$ into equivalence classes by their planes of motions as specified below:

1. Define $S^{1}:=S^{2}\left(\text{the sphere of }B of radius R\right)\cap\left(x,z\right)-plane$; for visualization, consider $S^{1}$ as a clock: $\left(0, 12\right] \left(o'clock\right)$. Then $9, 3\in EQ\equiv$the equator of $B$, of latitude $=0$; consider the restricted disk formed by $EQ$, $\hat{D}_{9,3}\equiv\left(EQ\cup\text{its interior}\right)-\left\{\left(0,y,0\right)\left|-R\leq y\leq R\right.\right\}$(reason for the restriction in (4) below); then any $p_{\alpha}\in\hat{D}_{9,3}$ of radius $r_{\alpha}\in\left(0,R\right]$ spirals counterclockwise around/toward $O_{B}\in the spinning axis of B$ within this latitude $=0$; at $t_{0}=1$, $p_{\alpha}$ finishes its $2\pi-rotation$with $O_{B}p_{\alpha}=r_{\alpha}/e$. As such, $\hat{D}_{9,3}$ constitutes an equivalence class that share a common spinning axis, the $z-axis$, which happens to be the spinning axis of $B$ and corresponds to $12\in S^{1}$. That is, an equivalence class is represented by its (perpendicular) spinning axis, and thus $12\in S^{1}$represents $\hat{D}_{9,3}$. In turn:
2. $1\in S^{1}$ represents $\hat{D}_{10,4}$, and
3. $2\in S^{1}$ represents $\hat{D}_{11,5}$.
4. Now, since the $z-axis$ is the spinning axis of $B$, any $p\in B\cap z-axis\equiv B_{z}$ must have a constant velocity toward $O_{B}$, i.e., of no spiral motions; by continuity, $D_{12,6}\supset B_{z}$ also have a constant velocity toward $O_{B}$, of no spiral motions. Following this divide by $D_{12,6}$:
5. $10\in S^{1}$ represents $\hat{D}_{1,7}$,
6. $11\in S^{1}$ represents $\hat{D}_{2,8}$, and
7. $12\in S^{1}$ represents $\hat{D}_{3,9}\equiv\hat{D}_{9,3}$, returning to (1).
